# FliL Functions in Diverse Microbes to Negatively Modulate Motor Output via Its N-Terminal Region

**DOI:** 10.1128/mbio.00283-23

**Published:** 2023-02-28

**Authors:** Xiaolin Liu, Anna Roujeinikova, Karen M. Ottemann

**Affiliations:** a Department of Microbiology and Environmental Toxicology, University of California, Santa Cruz, California, USA; b Infection and Immunity Program, Department of Microbiology and Department of Biochemistry and Molecular Biology, Monash Biomedicine Discovery Institute, Monash University, Clayton, Australia; University of Utah

**Keywords:** motor output, motility, flagella, nanomachine, motor, flagellar motility

## Abstract

The flagellar motor protein FliL is conserved across many microbes, but its exact role has been obscured by varying *fliL* mutant phenotypes. We reanalyzed results from *fliL* studies and found they utilized alleles that differed in the amount of N- and C-terminal regions that were retained. Alleles that retain the N-terminal cytoplasmic and transmembrane helix (TM) regions in the absence of the C-terminal periplasmic domain result in loss of motility, while alleles that completely lack the N-terminal region, independent of the periplasmic domain, retain motility. We then tested this prediction in Helicobacter pylori *fliL* and found support for the idea. This analysis suggests that FliL function may be more conserved across bacteria than previously thought, that it is not essential for motility, and that the N-terminal region has the negative ability to regulate motor function.

## OBSERVATION

Bacteria use flagella to locate their optimal environments, via swimming in liquid and swarming on wet surfaces. The flagellum is a complex ion-driven rotary nanomachine, comprised of many proteins. Some of these have poorly understood functions, a gap that prevents us from fully comprehending how this machine works.

Flagella rotate when ions move through the cytoplasmic membrane-embedded stator complexes, consisting of MotA and MotB, and generate torque (1, 2). This torque is applied to the flagellar rotor cytoplasmic ring, consisting of FliM, FliN, and FliG, making the rotor and the extracellular filament spin.

One protein of unknown function that is positioned within the torque/rotation part of the flagellum is FliL. This protein interacts with MotA, MotB, FliG, and the MS ring protein FliF (3). FliL is found in flagellated bacteria, but its function is mysterious. FliL has been suggested to be required for flagellar rotation, motor integrity, and/or surface sensing (4–6). The literature, however, is confusing because *fliL* deletion mutations are reported to have different and sometimes opposite phenotypes, ranging from nonmotile (5, 7–9), to slightly decreased motility or enhanced motility (4, 7). FliL is a single-span cytoplasmic membrane protein with a small cytoplasmic N-terminus, a transmembrane domain (TM), and a large extracytoplasmic C-terminal region, the fold of which resembles an stomatin/prohibitin/flotillin/HflK/C (SPFH) domain found in proteins involved in ion channel regulation (9, 10). The FliL extracytoplasmic domain forms a circle of rings, each coaxially sandwiched between MotA and the peptidoglycan-binding domain of MotB of a respective stator unit (9, 11). Overall, FliL appears to be important for flagellar motility, but it has been difficult to assess its exact role and whether it plays similar roles in diverse microbes. Here, we provide insight into this issue by identifying that previous work compared different types of *fliL* mutants. Our analysis suggests that a key variation is inclusion or exclusion of the N-terminal cytoplasmic and transmembrane regions. We support our ideas with a direct test in Helicobacter pylori. Our findings suggest that FliL is not essential for flagellar motility, but instead that the FliL N-terminal region acts as a motility inhibitor when retained without the extracytoplasmic C-terminal region.

**FIG 1:**
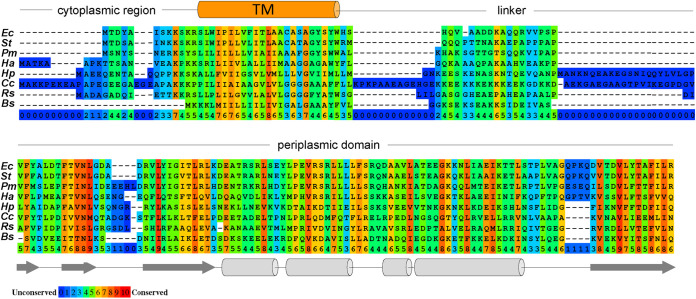
FliL proteins from different bacteria share a conserved secondary structure. Secondary structure guided sequence alignment of FliL proteins from Escherichia coli (*Ec*), Salmonella *typhimurium* (*St*), Proteus mirabilis (*Pm*), Herminiimonas arsenicoxydans (*Ha*), Helicobacter pylori (*Hp*), Caulobacter crescentus (*Cc*), Rhodobacter sphaeroides (*Rs*), and Bacillus subtilis (*Bs*). Conserved amino acids are highlighted in color, with red color/number 10 indicating higher conservation and dark blue color/number 0 indicating lower conservation. The predicted transmembrane (TM) helix is orange, the periplasmic domain is shaded in gray. The secondary structure shown under the sequences was derived from the crystal structure of the periplasmic domain of H. pylori FliL (9).

*fliL* mutant phenotypes are reported to vary between and even within the same bacterial species (4, 12, 13). To begin to understand these divergent phenotypes, we analyzed all published *fliL* mutants (Table S1). Our goal was to evaluate whether there were patterns to the type of *fliL* alleles and their phenotypes. We were not able to use all the reports, however, because in some cases the bacterial species had more than one flagellar system with each system encoding a FliL with unclear relations between them (10, 11, 14). Some reports suggested that the mutants were polar or otherwise non-complementable, or the reports lacked full motility data (Table S1). Our data set contained *fliL* mutants from eight species belonging to Alphaproteobacteria (Rhodobacter sphaeroides, Caulobacter crescentus [15]), Betaproteobacteria (Herminiimonas arsenicoxydans [16]), Gammaproteobacteria (Escherichia coli [4, 7, 12], Salmonella enterica serovar Typhimurium [7, 12], Proteus mirabilis [4]), Campylobacterota (Helicobacter pylori [9]), and Firmicutes (Bacillus subtilis [15, 17]) (Table S1). FliL proteins from these bacteria share a conserved secondary structure: a short, 2-28-residue N-terminal cytoplasmic region; an ~23-residue TM; a variable-length linker; and an ~200-residue C-terminal extracytoplasmic domain (Fig. 1). The conserved structure suggests that these FliL proteins perform similar functions.

10.1128/mbio.00283-23.1 TABLE S1 Description of all published *fliL* mutants, including a description of the mutation and phenotypes. Inclusion criteria is explained, with alleles included in our study indicated in green. Download Table S1, DOCX file, 0.06 MB. Copyright © 2023 Liu et al. 2023 Liu et al. https://creativecommons.org/licenses/by/4.0/ This content is distributed under the terms of the Creative Commons Attribution 4.0 International license.

**FIG 2:**
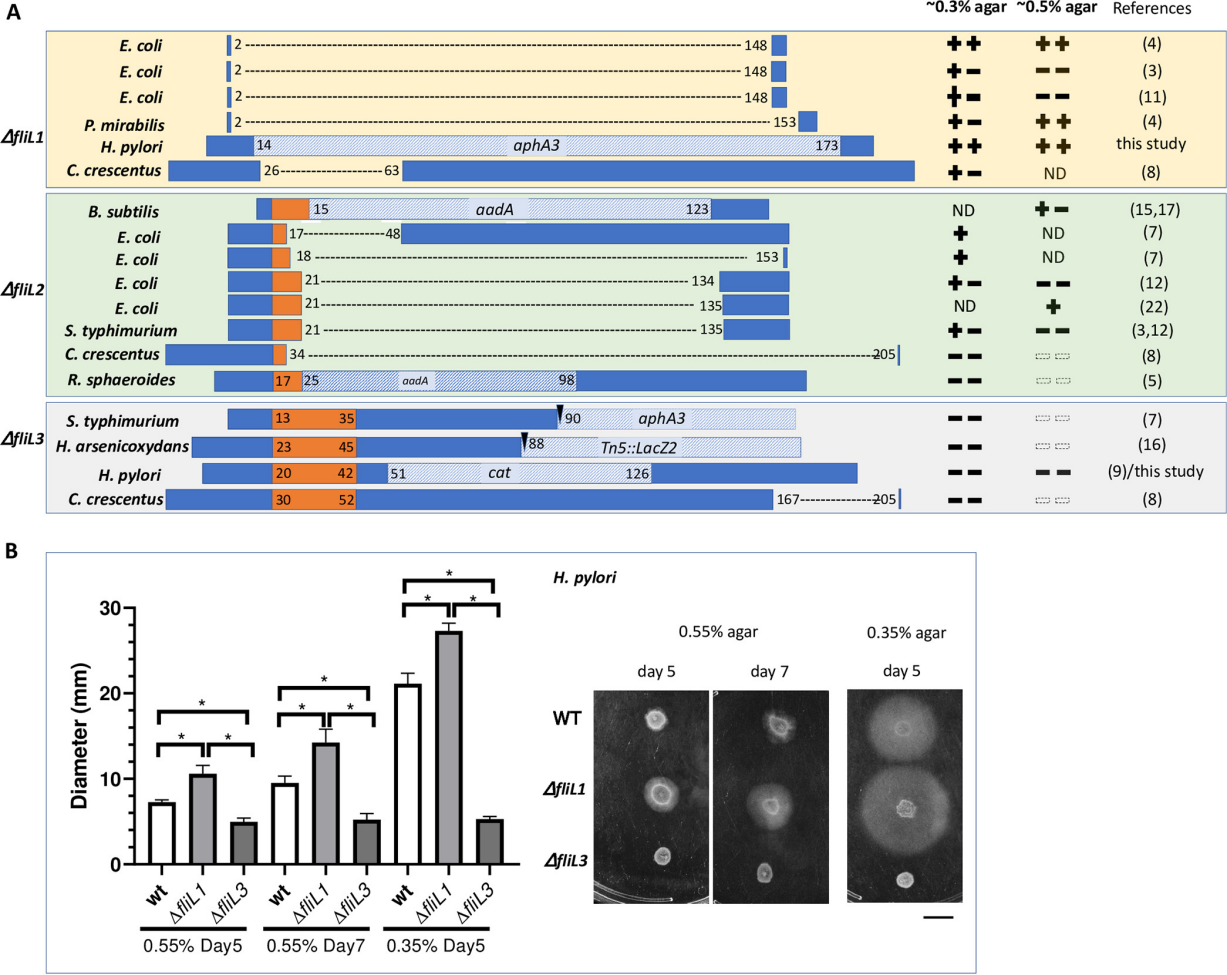
Comparison of phenotypes of strain with distinct *fliL* alleles on ~0.3% and ~0.5% agar. (A) Diagram of regions deleted in different *fliL* alleles and their cognate phenotypes on ~0.3% agar or ~0.5% agar. Dashed lines indicate deleted regions, any inserted antibiotic resistance genes are shown with oblique line patterns plus gene name, transmembrane region is colored orange. Amino acid residue numbers indicate region boundaries. Δ*fliL1* alleles lack the entire TM region; Δ*fliL2* alleles lack part of the TM region; Δ*fliL3* alleles retain the TM region. Phenotypes are given as, 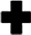 Migration similar to wild type (WT); 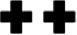 Migration greater than WT; 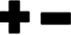 Migration less than WT; 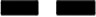 No migration; 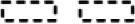. Predicted migration based on behavior on ~0.3% agar; ND, Not Determined. (B) H. pylori *fliL* mutant behavior on soft agar. Representative pictures and quantification of behavior of H. pylori WT, Δ*fliL1*, and Δ*fliL3* mutants on 0.55% and 0.35% agar plates, with scale bar = 1.0 cm. *n* = 4, error bars represent standard deviation, and * indicates *P* < 0.01 using Student's *t* test.

We then analyzed the details of the different Δ*fliL* mutants. We found that there was significant variation in the length of the N-terminal and C-terminal regions retained in the Δ*fliL* mutants. At the N-terminus, there were three types of variations: full loss of the TM (Δ*fliL1*); retention of the cytoplasmic region plus part of the TM (Δ*fliL2*); or retention of the cytoplasmic region plus the whole TM (Δ*fliL3*) (Fig. 2). At the C-terminus, the variation could be classified as complete loss, retention of about half of the FliL domain, or retention of a significant portion of the FliL and C-terminal domain (Fig. 2).

After classifying these types of *fliL* alleles, we then analyzed whether there were any patterns to *fliL* alleles that retained or lost motility on ~0.3% soft agar plates. One pattern immediately jumped out: alleles that deleted the entire TM (Δ*fliL1*) caused minimal motility defects, and in some cases even resulted in enhanced motility compared to wild type (Fig. 2A). In contrast, alleles that retained all of the TM (Δ*fliL3*) showed severe motility defects (Fig. 2A). Alleles with a partial TM (Δ*fliL2*) showed intermediate and variable motility phenotypes. In contrast to the N-terminal region, there was no obvious correlation with types of C-terminal mutations (Fig. 2A). These results suggest that flagellar motors can function without FliL if it is fully removed, but that retention of partial N-terminal FliL sequences results in loss of soft-agar migration.

Loss of movement on 0.3% soft agar plates could be due to defects in motility, chemotaxis, and/or growth. We thus examined the studies of Δ*fliL3* alleles to further explore the nature of the defects. Although the data are limited, motility was lost in the two analyzed for this ability, C. crescentus and H. pylori (8, 9). One S. enterica species was found to retain motility in liquid, which was slowed but had normal switching (7) (Table S1). Overall, these results suggest that retention of the FliL N-terminal region results in loss or slowed motility but does not affect switching.

The Δ*fliL* mutant previously constructed in H. pylori was a Δ*fliL3* allele and nonmotile (9) (Fig. 2A). We experimentally tested our hypothesis that the role of FliL in motility is associated with its TM by constructing an H. pylori Δ*fliL1* mutant lacking the entire TM (Fig. 2A). This mutant retained migration on soft agar (Fig. 2B). Indeed, the Δ*fliL1* mutant in H. pylori showed even greater soft agar migration than wild type (Fig. 2B). Overall, these results support the idea that *fliL* is not required for motility.

We were curious about the observation that the Δ*fliL1* allele showed elevated soft agar migration. Because FliL has been suggested to play a role in surface-associated responses, we examined *fliL* mutant phenotypes on soft-agar plates with high agar concentrations, between 0.5% and 1%, concentrations that support the surface-associated behavior called swarming (18). Δ*fliL1* mutants in E. coli (4) and P. mirabilis (4) migrated to a greater extent than their wild types (WTs) on ~0.5% soft agar (Fig. 2A). This response was similar to that of H. pylori (Fig. 2), suggesting the removal of *fliL* can result in motility that is more effective under elevated agar conditions. However, two other E. coli Δ*fliL1* alleles (3, 11), with deletions of the same regions as the allele above, were found to have high agar migration defects (Fig. 2A), indicating other unknown factors are associated with the function of FliL under high agar conditions.

Our analysis suggests that loss of *fliL* has a more consistent phenotype on ~0.3% soft agar across microbes than previously expected. FliL is not needed for flagellar motor function under this condition, and indeed, it appears to negatively regulate motility via its N-terminal region, including the TM helix. Our work suggests that future *fliL* alleles should be made with care to exclude the TM region if seeking a null allele. The idea that FliL N-terminal region exerts negative motor control is new, and it is not yet clear how this might occur. One idea comes from the observation that motility defects were restored in Δ*fliL3* mutants by extragenic suppressor mutations in the region of the *motB* gene corresponding to the plug (3, 5). Given that FliL and MotB are close to each other in the motor and interact *in vitro* (3, 9, 19), we propose that the FliL N-terminal region interacts with MotB so as to prevent plug opening, block ion flow, and inhibit motility. In WT FliL, because we found that alleles that retained the N-terminal region without the C-terminal one resulted in loss of motility, we propose that the C-terminal domain may act to regulate the N-terminal region.

Although our analysis did not contain bacteria with dual flagellar systems, the inhibiting function of the FliL N-terminus on motor output has also been reported in V. alginolyticus (10, 20), *B. diazoefficiens* (11), and V. fischeri (14). These studies show that Δ*fliL1* alleles retain motility in both polar or lateral systems, while Δ*fliL3* mutants show severe defects (Table S1). These combined results strongly support FliL is not an essential component of the flagellar motor, explaining why the *fliL* gene avoided detection by classical loss-of-function genetic analysis for a long time.

In addition to the differences in terms of what parts of *fliL* were deleted, we also noticed that *fliL* mutant phenotypes may be influenced by experimental conditions. For example, decreases in temperature alter the FliL soft-agar phenotype (4). In addition, the motility of Borrelia burgdorferi Δ*fliL* was tested by adding agarose instead of agar (6). These variations have not been systematically addressed, making it hard to compare results. Our improved understanding of *fliL* mutant construction will allow these variables to be studied.

FliL has been proposed to function in the surface-associated response in P. mirabilis (21), V. alginolyticus (10, 20), and *B. diazoefficiens* (11). The only direct evidence examining the function of FliL on mechanosensing comes from studies in E. coli (22, 23), which found there was no difference in force generation between WT and Δ*fliL* strains under external load. The two Δ*fliL* mutants (PL111 and PL62) used in these studies are *fliL2* types of alleles, a type with variable phenotypes (Fig. 2; Table S1), suggesting this result needs to be reexamined. Instead, the greater motile phenotypes of Δ*fliL1* on 0.5% agar plates suggest that FliL does have a function on motor output under viscosity. The stop or slow down of flagellar rotation in Δ*fliL3* suggests that the role of FliL on high viscosity might be achieved by affecting torque production, with the activity of the MotB ion channel regulated by the C-terminal FliL domain, which is similar to the function of the domain found in FliL, SPFH, in eukaryotic stomatin (10, 24, 25).

The information about regions deleted in the *fliL* mutants and cognate phenotypes on ~0.3% and ~0.5% agar plates was collected from published articles by May 2022 as cited in Table S1. The sequence alignment was performed using PRALINE (https://www.ibi.vu.nl/programs/pralinewww/) and edited in Jalview. The secondary structure and transmembrane regions were predicted using YASPIN and TMHMM (http://www.cbs.dtu.dk/services/TMHMM/), respectively. The Δ*fliL1* in H. pylori SS1 was constructed using natural transformation with a Δ*fliL::aphA3* that retained first 40 bp and the last 32 bp of *fliL* (primers available upon request). H. pylori motility on soft agar plates was tested using plates composed of Brucella broth, 2.5% (vol/vol) heat-inactivated fetal bovine serum (FBS), and either 0.35% or 0.55% Bacto agar. These plates were inoculated with H. pylori from overnight Brucella broth/10% FBS cultures adjusted to an OD_600_ of 0.15. Plates were incubated at 37°C, 10% CO_2_, 5% O_2_, 85% N_2_.
