# Correction for Liu et al., “FliL Functions in Diverse Microbes to Negatively Modulate Motor Output via Its N-Terminal Region”

**DOI:** 10.1128/mbio.02396-23

**Published:** 2023-10-24

**Authors:** Xiaolin Liu, Anna Roujeinikova, Karen M. Ottemann

## AUTHOR CORRECTION

Volume 14, no. 2, e00283-23, 2023, https://doi.org/10.1128/mbio.00283-23. It has been drawn to our attention that there is an error in the *Rhodobacter sphaeroides* FliL amino acid sequence used in this study. *R. sphaeroides* encodes two FliL proteins, and we should have used WP_002720207.1 rather than WP_002721881.1. Accordingly, there are errors in two figures as detailed below. These errors do not change the conclusions. We thank Dr. Laura Camarena for pointing out these errors and apologize for any inconvenience they may have caused.

Page 2, Fig. 1: There are errors in the sequence alignment. Figure 1 should appear as shown below.

**Fig 1:**
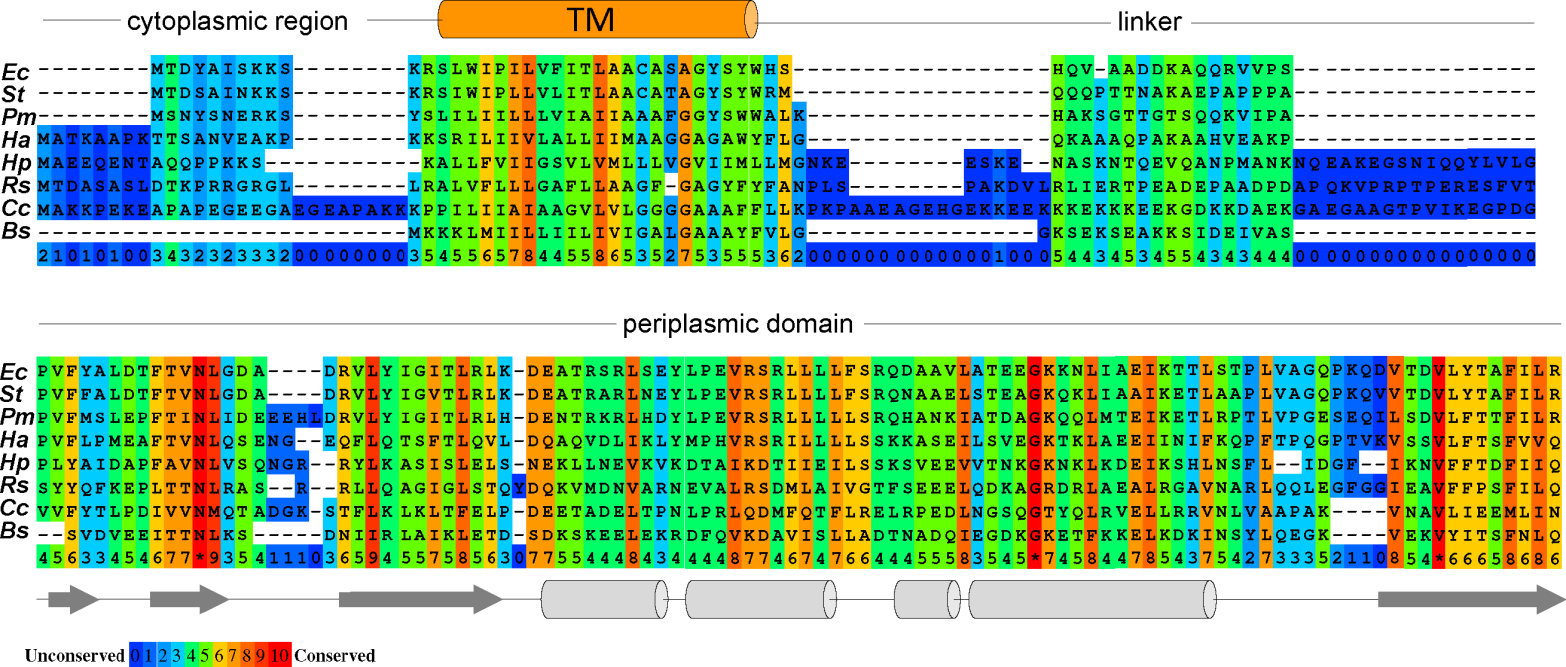


Page 3, Fig. 2A: There are errors in the region that was indicated as deleted in the *R. sphaeroides fliL* mutant. The residues that remain in the *R. sphaeroides* FliL transmembrane region, in the mutant, are amino acids 21 to 24. This remaining region is 4 residues and not 8 residues (17 to 24) as originally suggested. Figure 2A should appear as shown below.

**Fig 2:**
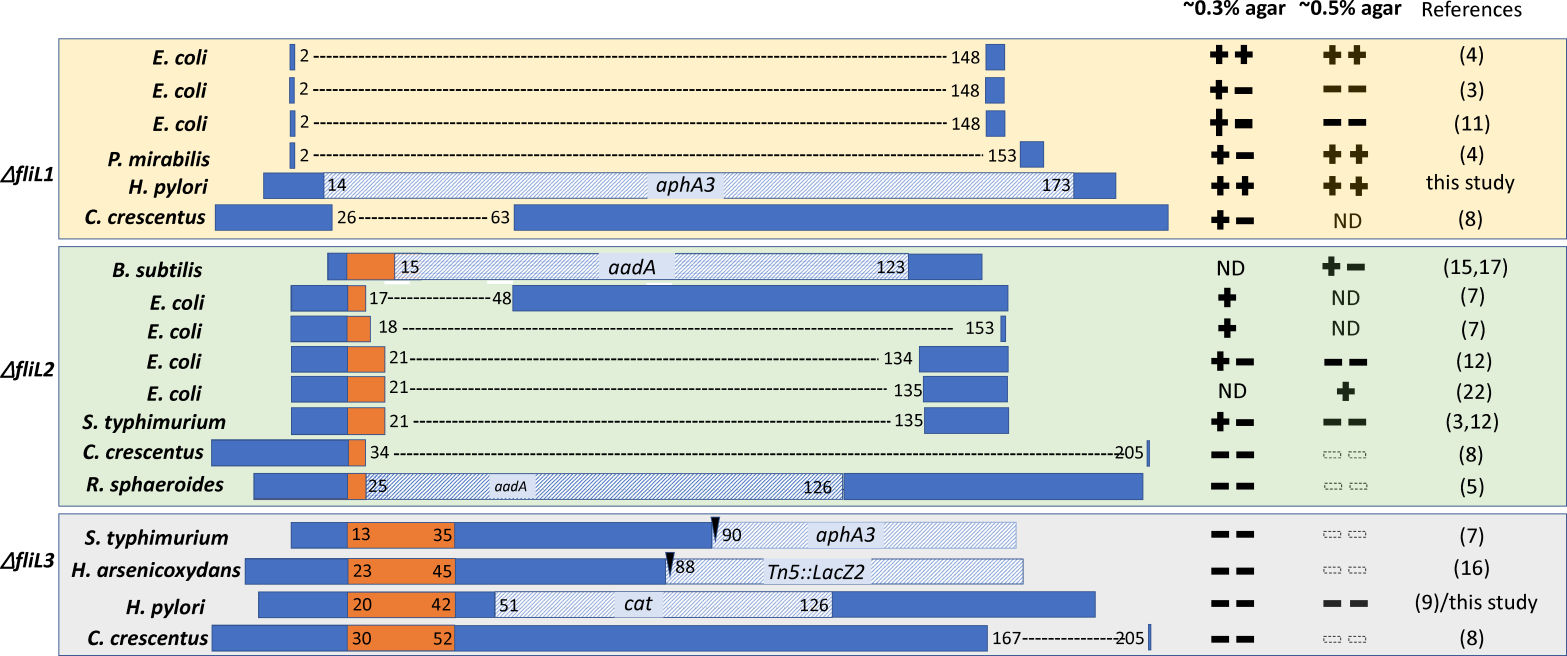


Supplemental material: We also noticed an error in Table S1; the *R. sphaeroides fliL* allele should be indicated as Δ*fliL2*. Revised supplemental material is included with this correction.

## SUPPLEMENTAL MATERIAL

Supplemental Table mbio.02396-23-s0001.docx Corrected Table S1.

ASM does not own the copyrights to Supplemental Material that may be linked to, or accessed through, an article. The authors have granted ASM a non-exclusive, world-wide license to publish the Supplemental Material files. Please contact the corresponding author directly for reuse.

