# Supplementary material for: FliL Functions in Diverse Microbes to Negatively Modulate Motor Output via Its N-Terminal Region
Source: mBio. 2023 Feb 28;14(2):e00283-23. doi: 10.1128/mbio.00283-23 (PMC10127578; doi:10.1128/mbio.00283-23)
Supplement: TABLE S1 [file mbio.00283-23-s0001.docx]

**Table S1**. Description of all published *fliL* mutants, including a description of the mutation and phenotypes. Inclusion criteria is explained, with alleles included in our study indicated in green.

| **Species and *fliL* allele** | **Strain name used in publication** | **Description of *fliL* deletion** | **Included and reason** | **Phenotype: In liquid swimming/rotation** | **Phenotype: Swimming (on ~0.3% agar plate)** | **Phenotype: Swarming (on ~0.5% agar plate)** | **Reference** |
| --- | --- | --- | --- | --- | --- | --- | --- |
| ***Bacillus subtilis*** |  |  |  |  |  |  |  |
| Δ*fliL2* | DS6540 Δ*fliL* | In-frame deletion of codon 15-123. | Included |  |  | +- | (1, 2) |
| ***Borrelia burgdorferi*** |  |  |  |  |  |  |  |
| Δ*fliL1* | Δ*fliL* | In-frame deletion of whole *fliL* gene. The *fliL* gene (gene locus BB0279, a 537-bp gene) was inactivated by replacing fliL with the aadA coding sequence for streptomycin-spectinomycin resistance | Not included, because in this study agarose was used to make soft-agar plates instead of agar, making comparison with other *fliL* mutants difficult. | defect | --  (Tested with agarose instead of agar) |  | (3) |
| ***Bradyrhizobium diazoefficiens*** |  |  |  |  |  |  |  |
| Δ*fliL1* | Δ*fliL_S_*, Subpolar *fliL* | In-frame deletion containing N-terminal 21 codons and C-terminal 11 codons. | Not included, because it is a dual flagellar system. |  | ++ | ND | (4) |
| Δ*fliL1* | Δ*fliL_L_*, Lateral *fliL* | In-frame deletion containing N-terminal 4 codons and C-terminal 13 codons. |  |  | +- | ND | (4) |
| ***Caulobacter crescentus*** |  |  |  |  |  |  |  |
| Δ*fliL1* | ΔL1 | In frame deletion that removed 38 internal codons from 26 to 63. | Included | motile | +- | ND | (5) |
| Δ*fliL2* | ΔL | In-frame deletion that retained only codons 1-33. | Included | Non-motile | -- | ND | (5) |
| Δ*fliL3* | ΔL2 | A deletion of the DNA sequence coding for the C-terminal 39 amino acids | Included | Non-motile | -- | ND | (5) |
| ***Escherichia coli*** |  |  |  |  |  |  |  |
| Δ*fliL1* | YL103 (RP437Δ*fliL*) | In-frame deletions of nt 4–444 (encoding residues 2–148) | Included, because the *fliL*1 mutant can be complemented. |  | ++ | ++ | (6) |
| Δ*fliL1* | JP633 (RP437Δ*fliL*) | in-frame deletions 4-444 (encoding residues 2–148) | Included, same deletion as in YL103 above (6). |  | +- | -- | (7) |
| Δ*fliL1* | JP1297 (MT02Δ*fliL*) | in-frame deletions of nt 4-444 (encoding residues 2–148) | Not included, same deletion as in YL103 above (6) and no data on soft-agar plates. |  | ND | ND | (7) |
| Δ*fliL1* | MG1655Δ*fliL* | in-frame deletions 4-444 (encoding residues 2–148) | Included |  | +- | -- | (4) |
| Δ*fliL2* | UA332 | In-frame deletion of nt 61–402 (encoding residues 21-134) | Included |  | +- | -- | (8) |
| Δ*fliL2* | EKK9 Δ(BamHI-ClaI) | in-frame deletions retaining only codons 1 to 17 and the last codon (codon 154) | Included |  | +- | ND | (9) |
| Δ*fliL2* | AW330 Δ(BamHI-MluI) | In-frame deletions of losing the codons 17 to 48 | Included |  | + | ND | (9) |
| Δ*fliL2* | PL111 | In-frame deletions of sequence from aa 21-135 | Included |  | ND | + | (10) |
| Δ*fliL2* | PL62 | In-frame deletions of sequence coding for aa 21–135 | Not included, because there is no data on soft-agar plates, and it is the same deletion as in PL111 above (10). |  | ND | ND | (11) |
| Δ*fliL* | JM7623 (Km^r^ insertion mutant) | insertion of a kanamycin resistance gene cassette, but insertion site location not clear | Not included, because this mutant has polar effect, and the kn cassette insertion site is unclear. |  |  |  | (9) |
| Δ*fliL* | MG1655 Δ*fliL* | In-frame deletions with unclear location description | Not included, because deleting region of this mutant is unclear. |  | +- | -- | (4) |
| ***Helicobacter pylori*** |  |  |  |  |  |  |  |
| Δ*fliL1* | Δ*fliL* | In-frame deletion of codon 14-173. | Included | Motile | ++ | ++ | This study |
| Δ*fliL3* | Δ*fliL* | In-frame deletion of codon 51-126. | Included | Non-motile | -- | -- | (12) |
| ***Herminiimonas arsenicoxydans*** |  |  |  |  |  |  |  |
| Δ*fliL3* | Δ*fliL* | In-frame insertion of a mini-Tn*5*::lacZ2 (*lacZ*-containing reporter gene transposon) in the 88th codon of the *fliL* gene | Included | ND | -- | ND | (13) |
| ***Proteus mirabilis*** |  |  |  |  |  |  |  |
| Δ*fliL1* | YL1006 | 90% of *fliL* deleted (YL1006 [*fliL*Δnt 4–459]) | Included |  | ++ | ++ | (6) |
| Δ*fliL1* | YL1003 | YL1003 (fliL::kan-nt 30), BB2000 with group II intron insertion in the nt 30 of *fliL*-Kn | Not included, because the phenotype of *fliL* itself cannot be deduced. Its phenotype was attributed to the presence of the C-terminal region of *fliL* DNA. |  | +- | -- | (14) |
| Δ*fliL3* | BB2204 | BB2000 *fliL*::Tn5-Cm; The insertion site of Tn5-CM in *fliL* was determined to be at base 432 of the *fliL* gene, 50 bases from the 3’ end of the coding sequence. | Not included, because this mutant has polar effect. |  | -- | -- | (15, 16) |
| ***Pseudomonas putida*** |  |  |  |  |  |  |  |
| Δ*fliL3* | Δ*fliL* | An kn insertion at the 50th residue. | Not included, because this mutant has polar effect with no flagella. |  | -- | ND | (17) |
| ***Rhodobacter sphaeroides*** |  |  |  |  |  |  |  |
| Δ*fliL3* | FS3 (*ΔfliL::aadA*) | The *ΔfliL3::aadA* allele was generated by cloning two independent PCR products, the first one containing 770 bp upstream from the stop codon of *fliK* and 72 bp downstream from the start codon of *fliL* and the second one containing 193 bp upstream from the stop codon of *fliL* and 839 bp downstream from the start codon of *fliM* | Included | ND | -- | ND | (18) |
| ***Salmonella typhimurium*** |  |  |  |  |  |  |  |
| Δ*fliL2* | UA74 | Derived from TH5111, An in-frame deletion of codon 21–135. | Included |  | +- | -- | (7, 8) |
| Δ*fliL3* | SJW2295 (frameshift mutant) | A single-base insertion at codon 90 of *fliL*, resulting in the loss of over 40% of the wild-type sequence | Included. | Less vigorously motile, flagella rotate slowly with reversals | -- | ND | (9) |
| ***Silicibacter sp. TM1040*** |  |  |  |  |  |  |  |
| Δ*fliL* | *fliL*::EZ-Tn5, Kan | Random transposon mutagenesis using EZ-Tn5  (R6Kori/KAN-2) no clear site information | Not included, because the Tn5 insertion site is not clear. |  | -- | ND | (19) |
| ***Vibrio alginolyticus*** |  |  |  |  |  |  |  |
| Δ*fliL1* | Polar *fliL* | Clear in-frame deletion | Not included, because it is a dual flagellar system. |  | +- | ND | (20) |
| Δ*fliL1* | Later *fliL* | Clear in-frame deletion |  |  | +- | -- | (21) |
| ***Vibrio fischeri*** |  |  |  |  |  |  |  |
| Δ*fliL3* | *fliL1* | Campbell-type (insertion-duplication) mutagenesis inserting region from 57th-122th aa | Not included, because it is a dual flagellar system. |  | -- | ND | (22) |
| Δ*fliL3* | *fliL2* | Campbell-type mutagenesis inserting region from 34th-100th |  |  | -- | ND | (22) |

1. Hall AN, Subramanian S, Oshiro RT, Canzoneri AK, Kearns DB, O'Toole G. 2018. SwrD (YlzI) promotes swarming in *Bacillus subtilis* by increasing power to flagellar motors. Journal of Bacteriology 200:e00529-17.

2. Calvo RA, Kearns DB, O'Toole GA. 2015. FlgM is secreted by the flagellar export apparatus in *Bacillus subtilis*. Journal of Bacteriology 197:81-91.

3. Motaleb MA, Pitzer JE, Sultan SZ, Liu J. 2011. A novel gene inactivation system reveals altered periplasmic flagellar orientation in a *Borrelia burgdorferi flil* mutant. Journal of Bacteriology 193:3324-3331.

4. Mengucci F, Dardis C, Mongiardini EJ, Althabegoiti MJ, Partridge JD, Kojima S, Homma M, Quelas JI, Lodeiro AR. 2020. Characterization of FliL proteins in *Bradyrhizobium diazoefficiens*: lateral FliL supports swimming motility, and subpolar FliL modulates the lateral flagellar system. Journal of Bacteriology 202.

5. Jenal U, White J, Shapiro L. 1994. *Caulobacter* flagellar function, but not assembly, requires FliL, a non-polarly localized membrane protein present in all cell types. J Mol Biol 243:227-44.

6. Lee Y-Y, Belas R. 2015. Loss of FliL alters *Proteus mirabilis* surface sensing and temperature-dependent swarming. Journal of Bacteriology 197:159-173.

7. Partridge JD, Nieto V, Harshey RM. 2015. A new player at the flagellar motor: FliL controls both motor output and bias. mBio 6:e02367-14.

8. Attmannspacher U, Scharf BE, Harshey RM. 2008. FliL is essential for swarming: motor rotation in absence of FliL fractures the flagellar rod in swarmer cells of Salmonella enterica. Molecular Microbiology 68:328-341.

9. Raha M, Sockett H, Macnab RM. 1994. Characterization of the *fliL* gene in the flagellar regulon of *Escherichia coli* and *Salmonella typhimurium*. Journal of Bacteriology 176:2308-2311.

10. Chawla R, Ford KM, Lele PP. 2017. Torque, but not FliL, regulates mechanosensitive flagellar motor-function. Scientific Reports 7.

11. Lele PP, Hosu BG, Berg HC. 2013. Dynamics of mechanosensing in the bacterial flagellar motor. Proceedings of the National Academy of Sciences 110:11839-11844.

12. Tachiyama S, Chan KL, Liu X, Hathroubi S, Li W, Peterson B, Khan MF, Ottemann KM, Liu J, Roujeinikova A. 2022. The flagellar motor protein FliL forms a scaffold of circumferentially positioned rings required for stator activation. Proceedings of the National Academy of Sciences 119:e2118401119.

13. Muller D, Médigue C, Koechler S, Barbe V, Barakat M, Talla E, Bonnefoy V, Krin E, Arsène-Ploetze F, Carapito C, Chandler M, Cournoyer B, Cruveiller S, Dossat C, Duval S, Heymann M, Leize E, Lieutaud A, Lièvremont D, Makita Y, Mangenot S, Nitschke W, Ortet P, Perdrial N, Schoepp B, Siguier P, Simeonova DD, Rouy Z, Segurens B, Turlin E, Vallenet D, Dorsselaer AV, Weiss S, Weissenbach J, Lett M-C, Danchin A, Bertin PN. 2007. A tale of two oxidation states: bacterial colonization of arsenic-rich environments. PLoS Genetics 3:e53.

14. Lee YY, Patellis J, Belas R. 2013. Activity of *Proteus mirabilis* FliL is viscosity dependent and requires extragenic DNA. Journal of Bacteriology 195:823-832.

15. Cusick K, Lee Y-Y, Youchak B, Belas R. 2012. Perturbation of FliL interferes with *Proteus mirabilis* swarmer cell gene expression and differentiation. Journal of Bacteriology 194:437-447.

16. Belas R, Suvanasuthi R. 2005. The ability of *Proteus mirabilis* to sense surfaces and regulate virulence gene expression involves FliL, a flagellar basal body protein. Journal of Bacteriology 187:6789-6803.

17. Segura A, Duque E, Hurtado A, Ramos JL. 2001. Mutations in genes involved in the flagellar export apparatus of the solvent-tolerant *Pseudomonas putida* DOT-T1E strain impair motility and lead to hypersensitivity to toluene shocks. Journal of Bacteriology 183:4127-4133.

18. Suaste-Olmos F, Domenzain C, Mireles-Rodríguez JC, Poggio S, Osorio A, Dreyfus G, Camarena L. 2010. The flagellar protein FliL is essential for swimming in *Rhodobacter sphaeroides*. Journal of Bacteriology 192:6230-6239.

19. Belas R, Horikawa E, Aizawa S-I, Suvanasuthi R. 2009. Genetic determinants of *Silicibacter sp.* TM1040 motility. Journal of Bacteriology 191:4502-4512.

20. Zhu S, Kumar A, Kojima S, Homma M. 2015. FliL associates with the stator to support torque generation of the sodium‐driven polar flagellar motor of *V. ibrio*. Molecular Microbiology 98:101-110.

21. Takekawa N, Isumi M, Terashima H, Zhu S, Nishino Y, Sakuma M, Kojima S, Homma M, Imada K. 2019. Structure of *Vibrio* FliL, a new stomatin-like protein that assists the bacterial flagellar motor function. mBio 10:e00292-19.

22. Brennan CA, Mandel MJ, Gyllborg MC, Thomasgard KA, Ruby EG. 2013. Genetic determinants of swimming motility in the squid light‐organ symbiont Vibrio fischeri. MicrobiologyOpen 2:576-594.
